# ASSOCIATIONS BETWEEN MEASURES OF HEALTH AND OBJECTIVE AND SUBJECTIVE NEIGHBORHOOD QUALITY

**DOI:** 10.1093/geroni/igae098.0247

**Published:** 2024-12-31

**Authors:** Alexa Allan, Roland Thorpe, Jr., Orfeu Buxton, Lesley Ross, May Beydoun, Alan Zonderman, Michele Evans, Alyssa Gamaldo

**Affiliations:** The Pennsylvania State University, University Park, Pennsylvania, United States; Johns Hopkins University, Baltimore, Maryland, United States; The Pennsylvania State University, University Park, Pennsylvania, United States; Clemson University, Clemson, South Carolina, United States; National Institute on Aging, Baltimore, Maryland, United States; National Institute on Aging, Baltimore, Maryland, United States; National Institute on Aging, Baltimore, Maryland, United States; Clemson University, Clemson, South Carolina, United States

## Abstract

The current study examined the cross-sectional associations between self-reported mental/emotional (e.g., patient health questionnaire), sleep (e.g., Pittsburgh Sleep Quality Index), physical (e.g., medical conditions), and cognitive health (e.g., Montreal Cognitive Assessment) and subjective and objective measures of neighborhood quality. This preliminary analysis included 77 community-dwelling socioeconomically diverse Black and White adults (Mage = 62.17, SDage = 9.71; 70% female) from the HANDLSleep study. Neighborhood quality was assessed using self-reported measures of physical built disorder (e.g., graffiti), social cohesion (e.g., close-knit neighborhood), and social control (e.g., neighbors act if children disrespecting an adult). Area Deprivation Index (ADI) National and State values were extracted from the Neighborhood Atlas using participant addresses. Multivariable linear regression analyses were conducted using health measures as the outcome of interest. Models were adjusted for age, sex, race, education, and poverty status. In the adjusted models, participants living in neighborhoods of greater deprivation (i.e., State ADI) reported higher levels of depressive symptomology (p <.05) and worse sleep quality (p <.05). Likewise, higher reports of physical built disorder were associated with poorer cognition (i.e., attention, p <.05). Contrastingly, reports of better social cohesion were associated with lower insomnia severity scores (p <.05) and better performance on a measure of executive function (p <.01). Reports of more social control was also associated with better executive function (p <.05). These findings highlight the importance of examining both objective and subjective neighborhood characteristics as they pertain to different dimensions of health.

